# Using a Mixed Model to Evaluate Job Satisfaction in High-Tech Industries

**DOI:** 10.1371/journal.pone.0154071

**Published:** 2016-05-03

**Authors:** Sang-Bing Tsai, Chih-Yao Huang, Cheng-Kuang Wang, Quan Chen, Jingzhou Pan, Ge Wang, Jingan Wang, Ta-Chia Chin, Li-Chung Chang

**Affiliations:** 1 Zhongshan Institute, University of Electronic Science and Technology of China, Guangdong, 528402, China; 2 College of Business Administration, Dongguan University of Technology, Guangdong, 523808, China; 3 Law School, Nankai University, Tianjin, 300071, China; 4 School of Business, Dalian University of Technology, PANJIN, 124221, China; 5 Business School, Nankai University, Tianjin, 300071, China; 6 College of Management and Economics, Tianjin University, Tianjin, 300072, China; 7 Management School, Tianjin Polytechnic University, Tianjin, 300387, China; 8 School of Management, Hangzhou Dianzi University, 310018, Zhejiang, China; 9 Guangzhou Vocational College of Science and Technology, Guangzhou, 510550, China; Southwest University, CHINA

## Abstract

R&D professionals are the impetus behind technological innovation, and their competitiveness and capability drive the growth of a company. However, high-tech industries have a chronic shortage of such indispensable professionals. Accordingly, reducing R&D personnel turnover has become a major human resource management challenge facing innovative companies. This study combined importance–performance analysis (IPA) with the decision-making trial and evaluation laboratory (DEMATEL) method to propose an IPA–DEMATEL model. Establishing this model involved three steps. First, an IPA was conducted to measure the importance of and satisfaction gained from job satisfaction criteria. Second, the DEMATEL method was used to determine the causal relationships of and interactive influence among the criteria. Third, a criteria model was constructed to evaluate job satisfaction of high-tech R&D personnel. On the basis of the findings, managerial suggestions are proposed.

## Introduction

R&D activity is widely considered a crucial indicator of a country's technological capability. In turn, technological development is used as a measure of competitiveness, economic development, and social progress at the national level. To gain an edge in a rapidly changing and increasingly globalized and competitive world, governments in developed countries dedicate themselves to promoting high-tech industries. In this context, technological R&D has received widespread attention, placing R&D professionals at the forefront of generating profits at the firm level and improving competitiveness at the industrial level.

Labor shortages, limited access to technical information, and high turnover rates are common hindrances of R&D progress and lead to insufficient capital. R&D is the core of the innovation mechanism of a company; thus, inadequate R&D personnel can limit the development of high-tech industries [1–2].

Because the innovative capability of R&D personnel is vital to achieving sustainability and maintaining product competitiveness on the cutting edge of the market, exploring R&D personnel’s job satisfaction and creating a proper work environment to retain such workers should be a top priority for human resource personnel in high-tech industries.

Job satisfaction has been widely discussed among scholars and managers [3]. In today’s business community, where human resources are treated as an indispensable corporate asset, maintaining and increasing job satisfaction, retaining competent workers, reducing turnover, improving the company’s overall work efficiency, appointing suitable personnel to suitable positions, and motivating employees to their full potential are all major tasks deliberated and performed by management.

This study aimed to develop criteria for evaluating the job satisfaction of high-tech R&D personnel. Importance–performance analysis (IPA) and the decision-making trial and evaluation laboratory (DEMATEL) method were combined to yield an IPA–fuzzy DEMATEL model. IPA was conducted to measure the importance of and satisfaction gained from the aforementioned criteria, and the fuzzy DEMATEL method was used to explore the causal relationships and interactive influence among the criteria. Finally, a criteria model was constructed to evaluate the job satisfaction of high-tech R&D personnel. This paper concludes by proposing managerial suggestions.

## Literature Review

### Characteristics of high-tech industries and R&D activity

High-tech industries are broadly classified as follows: (1) An industry can be “industry-based,” as defined by the proportion of R&D expenditure to the gross output value (or overall revenue) and the proportion of R&D employees to the total workforce of an industry. (2) An industry can be “product-based,” as defined by the average proportion of R&D expenditure to average overall revenue of a company. (3) Finally, an industry can be “industry- and product-based,” as defined by indicators such as having huge market potential, extensive interindustry relationships, high value-added products, high technical levels, low pollution, and low energy dependence [4–5].

Previous studies have identified some of the characteristics of high-tech industries: having advanced knowledge and technological intensiveness, a high proportion of R&D expenditure and technological personnel, and network externality; developing small products with high added-values; facilitating the reorganization of the existing industry structure; and producing products with short life cycles [6–7].

This study proposes the following high-tech industry characteristics:

1. Within high-tech industries, R&D expenditure accounts for a large share of the total operating costs.
2. Because intelligence is instrumental in the development of high-tech industries, most high-tech companies are small and medium-sized enterprises whose company size enables flexibility and adaptation in response to the rapid market changes of the industries.
3. Because high-tech industries develop products with high added-values established by innovative input, the locations of these industries are characteristically footloose.
4. High-tech industries are typically in close partnership with the academic community (especially with the higher education sector). Thus, combining the basic research capability of universities and the applied research capability of private firms can contribute to the industries’ development.
5. The most crucial component of production costs for high-tech industries is information access, instead of remuneration, or as conventionally discussed by location theory, transportation costs. This component is the main concern addressed during national policymaking on the industries’ development.

In an environment characterized by developing technologies, high uncertainty, rapid change, and increasingly shorter product life cycles, no company can survive or thrive with its existing products or services unless it consistently innovates in response [8].

High-tech industries are capital, technology, and knowledge intensive and emphasize innovation speed. Only by continually upgrading and improving technologies can they maintain their competitiveness. Consistent innovation entails the contribution of R&D personnel, whose competitiveness and capability are essential for a company’s growth. Innovation is required for competing in an increasingly competitive market, and it is R&D personnel who determine a company’s progression.

### Employee job satisfaction and job satisfaction criteria

Job satisfaction, or occupational contentedness, was first proposed by Hoppock [9], who defined job satisfaction as employees’ psychological and physiological satisfaction with environmental circumstances; it indicates their subjective reaction to the work environment. Since Hoppock developed the concept of job satisfaction, numerous studies have explored this topic. Vroom’s definition of job satisfaction emphasizes the degree of employees’ satisfaction or dissatisfaction with the job roles they perform: If the roles suit their orientations, they obtain job satisfaction [10].

Job satisfaction may also be affected by job-inherent factors that involve an employee’s objectives and expectations associated with the job [11]. In other words, the level of job satisfaction may be determined by the comparison between job outcomes and job objectives or expectations. Thus, according to Locke’s [12] discrepancy theory, an employee’s job satisfaction increases as the content, process, and outcomes of an actual job increasingly adhere to an employee’s objectives or expectations associated with the task. By contrast, if the task content, process, and outcome are distant from an employee’s objectives, the discrepancy can cause a psychological conflict, or cognitive dissonance, that in turn decreases job satisfaction.

In addition, personal and organizational factors can affect job satisfaction. Personal factors include demographic characteristics, capability, perception, cognitive ability, expectations, a sense of achievement, and personality traits [13–15]. Organizational factors can influence job satisfaction as well; these include compensation policy, work conditions, job rank, coworker relationships, supervisor–subordinate relationships, job security, promotion policy, required responsibility, and the possibility of career development [11,15,16].

In summary, the level of job satisfaction influences an employee’s behavior and attitudes toward the job he or she performs and directly affects job performance [17–18]. High job satisfaction can contribute to low absent-without-leave rates and strong intentions to stay and produce positive effects on the organization. By contrast, low job satisfaction can result in high absence rates and frequent employee turnovers.

Based on the studies discussed, this study defined job satisfaction as a person’s overall attitude toward his or her job and examined this attitude by using 10 satisfaction criteria: compensation, promotion, supervisors, fringe benefits, recognition, work environment, regulations and policies, coworkers, job nature, and communication.

## Method

The study was reviewed and approved by an institutional review board at the Zhongshan Institute, University of Electronic Science and Technology of China (ethics committee).

This study identified two limitations in job satisfaction criteria applied by previous studies. First, the importance and performance of each criterion have not been estimated in some studies; thus, their relevance could not be measured. Second, most studies have assumed that these criteria are independent of each other and no interactive influence or causal relationship exists between them. This assumption may limit the improvement of employee satisfaction criteria.

To address both limitations, this study integrated IPA and the fuzzy DEMATEL method to develop an IPA–fuzzy DEMATEL model. IPA was conducted first to measure the importance of and satisfaction gained from all job satisfaction criteria. The DEMATEL method was then used to determine the causal relationships and degrees of influence among the criteria. Finally, a criteria model was constructed to evaluate the job satisfaction of high-tech R&D personnel.

IPA estimates the mean perceived importance and performance of criteria, graphically displaying the results on a two-dimensional matrix [19–20]. The DEMATEL method addresses complex systems by establishing the interdependence among criteria in the systems and involves using matrix operations to estimate direct and indirect causal relationships and effects among these criteria. This method transforms the systems into causal relationships with definite structures, simplifying all criteria to causes and effects for measuring their influence on each other, which facilitates identifying core problems and improvement approaches within the systems [21–23].

Based on the aforementioned studies on high-tech industry characteristics and employee satisfaction criteria, this study developed a criteria model for evaluating the job satisfaction of high-tech R&D personnel. The model comprised 10 criteria: compensation, promotion, supervisors, fringe benefits, recognition, work environment, regulations and policies, coworkers, job nature, and communication.

### IPA model

**Fig 1:**
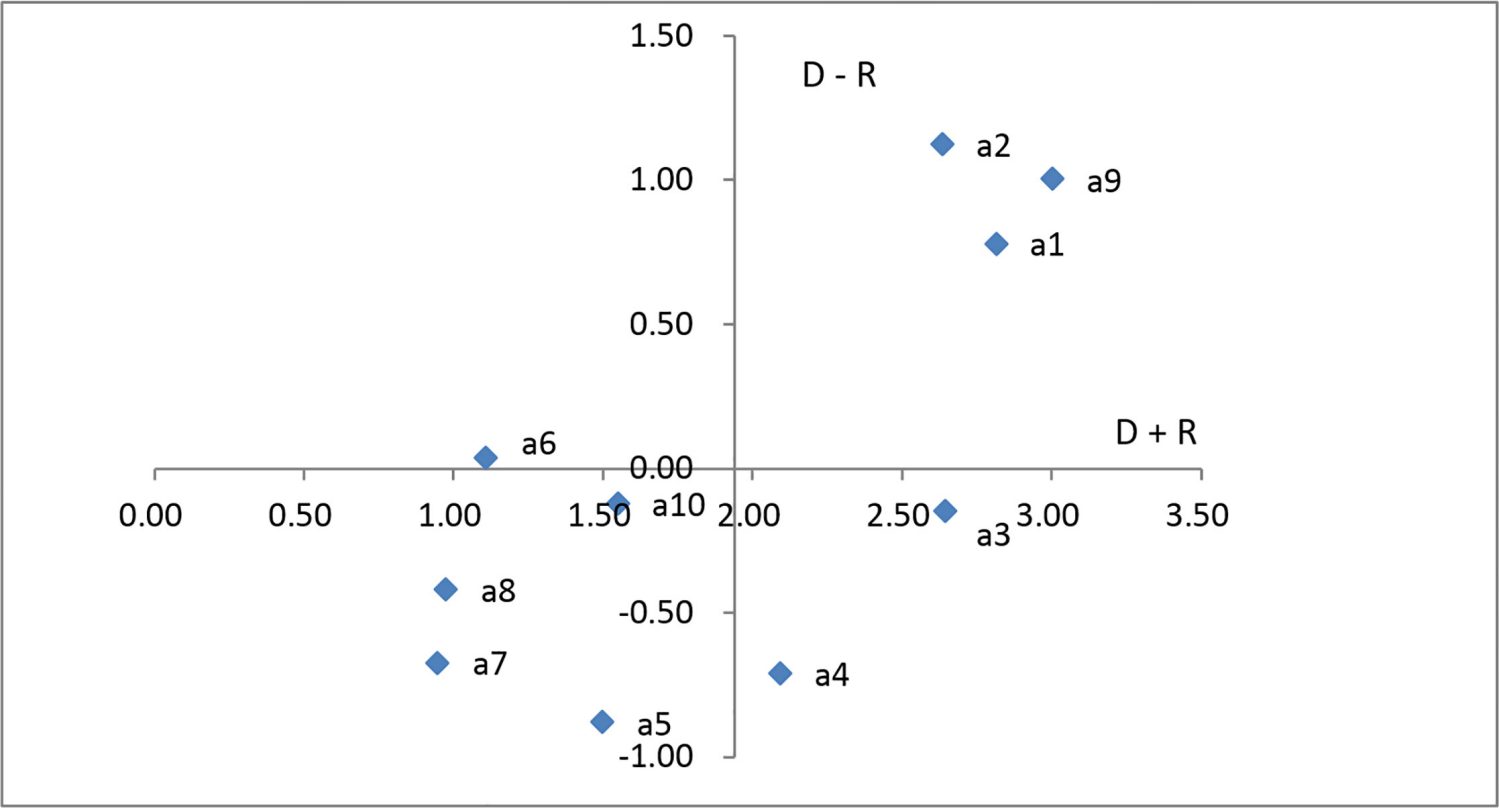
Importance–performance grid.

Martilla and James [24] first proposed and applied the framework of IPA. IPA displays the mean values of importance and performance service attributes on a two-dimensional grid with importance on the y-axis and performance on the x-axis (Fig 1).

The importance–performance grid comprises four quadrants:

1. Keep up the Good Work: Criteria that have high importance and provide high satisfaction. Criteria falling in this quadrant are categorized as “Keep up the Good Work.”
2. Concentrate Here: Criteria that have high importance but provide low satisfaction. Criteria falling in this quadrant are categorized as “Concentrate Here.”
3. Low Priority: Criteria that have low importance and provide low satisfaction. Criteria falling in this quadrant are categorized as “Low Priority.”
4. Possible Overkill: Criteria that have low importance but provide high satisfaction. Criteria falling in this quadrant are categorized as “Possible Overkill.”

Using this four-quadrant grid, managers can determine the effective use of limited resources and prioritize criteria for improvement to increase rater satisfaction.

### Decision-making trial and evaluation laboratory model

The DEMATEL method originated from the Natural Sciences and Humanities Research Plan proposed by the Battelle Institute in 1971, and was employed to explore complex world problems during the initial stage of its development, such as racial issues, starvation, environmental protection, and energy consumption. At that time, it was applied primarily to undertake three research topics: (1) the structures of world problems; (2) the analysis of complex world problems and development of adaptive solutions for the problems; and (3) the review of research, methodology, and data related to world problems [22, 25–27].

Lee et al. [28] suggested that the DEMATEL method identifies the causal relationships between factors and their effects on each other through a matrix operation, creating a causal diagram to examine the nature of a complex system and identify core problems and improvement approaches.

To conduct the DEMATEL method, analysis elements should satisfy three criteria [29–30]:

1. The nature of the problem is clearly defined: When a problem is being formulated, its nature must be defined to formulate correct solutions.
2. The degree of relationships of the problem is determined: The degree of the relationship of one problem with the others should be determined, with the strength of their relationship denoted by numbers such as 0, 1, 2, 3, or 4.
3. The characteristics of each problem element are identified: After the characteristics of all problem elements are identified, details (including affirmative and negative perspectives) about the problems should be provided.

The estimation procedure of the DEMATEL model is described as follows:

**Step 1: Establish a measurement scale and determine the levels of causal relationships between factors.** A literature review, the brainstorming process, and the expert-opinion method are conducted to list and define the factors that influence a complex system. Next, a scale measuring the levels of influence is designed for conducting pairwise comparison of factors, thereby determining the levels of their causal relationships.

**Step 2: Establish a direct-relation matrix.** After determining the meanings of the measurement scale, researchers administer questionnaires to experts, inviting them to conduct a pairwise comparison of the factors in accordance with their causal relationships and levels of interactive influence. Finally, a direct-relation matrix is formed, in which each value represents a level of interactive influence between two factors, and the values along the diagonal of the matrix are designated as 0.

$$X=\left[\begin{array}{cccc} 0 & x_{12} & \cdots & x_{1n}\\ x_{21} & 0 & \cdots & x_{2n}\\ \vdots & \vdots & \ddots & \vdots \\ x_{n1} & x_{n2} & \cdots & 0 \end{array}\right]$$ (1)

**Step 3: Calculate the normalized direct-relation matrix by using column vectors with maximal values as normalization baselines.**

$$\lambda =\frac{1}{\begin{array}{c} Max\\ 1\leq i\leq n \end{array}\left(\sum_{j=1}^{n}x_{ij}\right)},$$ (2)

N=λX. (3)

**Step 4: Calculate the direct/indirect-relation matrix T (namely, the total-relation matrix).**

$$T=\underset{k\to \infty }{\lim}\left(N+N^{2}+\cdots +N^{k}\right)=N{\left(I-N\right)}^{-1}$$ (4)

where *I* denotes the identity matrix.

**Step 5: Calculate the sum of the values of each column and each row.** The values of each column and row in the total-relation matrix *T* are summed. Next, *D*_*i*_ is designated as the sum of Row *i* and *R*_*j*_ as the sum of Column_*j*_. Consequently, both *D*_*i*_ and *R*_*j*_ involve indirect and direct influence.

$$D_{i}=\sum_{j=1}^{n}t_{ij}\begin{array}{cc} & \left(i=1,2,\ldots ,n\right) \end{array}$$ (5)

$$R_{i}=\sum_{i=1}^{n}t_{ij}\;(j=1,2,\ldots ,n).$$ (6)

**Step 6: Draw a DEMATEL causal diagram.** The sum of (*D + R*) is defined as the *prominence*, whereas in *k = i = j = 1*,*2*,…*n*, Criterion *k* signifies the sum of the influence of the criterion on other criteria, as well as their influence on it, from which the level of importance of Criterion *k* in the problems can be determined. The difference of (*D − R*) is defined as the *relation*, which refers to the difference between the influence of Criterion *k* on other criteria and their influence on it, from which the criterion’s levels of causal relationships in all of the problems can be identified. A positive value for (*D − R*) indicates that the criterion is more a cause than an effect, whereas a negative value represents the opposite. A causal diagram is drawn with (*D + R*) on the x-axis and (*D − R*) on the y-axis, simplifying complex causal relationships to intelligible visual structures. Using this visual information, decision-makers can discern the category of each factor according to its position on the diagram and formulate appropriate problem-solving decisions according to its level of influence.

If the value for (*D*_*k*_ *− R*_*k*_) is positive, Criterion *k* is categorized as a cause; if the value for (*D*_*k*_ *− R*_*k*_) is negative, Criterion *k* is categorized as an effect. A higher value of (*D*_*k*_ *+ R*_*k*_) suggests that the criterion’s influence on other criteria is greater than their influence on it. Based on the coordinate locations of (*D*_*k*_ + *R*_*k*_) and (*D*_*k*_ *− R*_*k*_), four attributes of the criteria are identified:

1. Positive (*Dk − Rk*) and high (*Dk + Rk*): The criterion is a cause of and driver for problem solving.
2. Positive (*Dk − Rk*) and low (*Dk + Rk*): The criterion is independent and can influence only a few other criteria.
3. Negative (*Dk − Rk*) and low (*Dk + Rk*): The criterion is independent and can be influenced by only a few other criteria.
4. Negative (*Dk − Rk*) and high (*Dk + Rk*): The criterion is the core problem that must be solved. However, because of its effect-category attributes, it cannot be directly improved.

## Results and Discussion

### Questionnaire administration

We propose criteria for evaluating the job satisfaction of R&D professionals in the liquid crystal display (LCD) industry. The evaluation results can be used to improve their satisfaction at the workplace. The criteria are as follows: compensation (a1), promotion (a2), supervisors (a3), fringe benefits (a4), recognition (a5), work environment (a6), regulations and policies (a7), coworkers (a8), job nature (a9), and communication (a10).

Two questionnaires were used. One was an IPA questionnaire comprising closed-ended questions regarding the importance and performance of the criteria scored using a 9-point Likert scale. The questions were rated on a scale from 1 (*highly dissatisfied*) to 9 (*highly satisfied*). This questionnaire was administered on November 16–30, 2015 to five academics, five general managers, and five associate HR managers in the LCD industry to derive expert opinions for developing R&D personnel job satisfaction criteria. Fifteen IPA questionnaires were distributed, with 15 valid responses for a return rate of 100%.

The second applied questionnaire was a DEMATEL questionnaire measured on a 10-point scale, with 9 denoting *the highest influence* and 0 denoting *no influence*. The questionnaire was administered to the same 15 experts on December 1–18, 2015. All the respondents were visited, briefed on the content of the questionnaire, and asked to complete the questionnaire. Fifteen valid responses were obtained with a return rate of 100%.

### IPA results

The average importance score (6.88) and performance score (6.64) were used as the baselines to determine the importance of each criterion as “high” or “low” and the performance of each criterion as “good” or “poor.” Management strategies for all criteria were subsequently identified.

The IPA results showed the importance and performance scores of the 10 R&D personnel job satisfaction criteria for the LCD industry, as provided by the invited experts (Table 1). The experts attributed high importance to promotion (a2), work environment (a6), and job nature (a9) and were satisfied with their performance. Thus, these three criteria were located in the Keep up the Good Work quadrant. The experts attributed high importance to compensation (a1) and fringe benefits (a4) but were dissatisfied with their performance. Thus, these two criteria were located in the Concentrate Here quadrant. The experts attributed low importance to regulations and policies (a7), coworkers (a8), and communication (a10) but were dissatisfied with their performance. Thus, these three criteria were located in the Low Priority quadrant. The experts attributed low importance to supervisors (a3) and recognition (a5) but were satisfied with their performance. Thus, these two criteria were located in the Possible Overkill quadrant.

**Table 1:** Importance and performance of R&D personnel job satisfaction criteria.

Item	Criteria	Importance	Performance	Management Strategy
a1	Compensation	7.8	6.2	Concentrate Here
a2	Promotion	7.5	7.3	Keep up the Good Work
a3	Supervisors	6.7	6.8	Possible Overkill
a4	Fringe benefits	6.9	6.5	Concentrate Here
a5	Recognition	6.7	6.8	Possible Overkill
a6	Work environment	6.9	6.9	Keep up the Good Work
a7	Regulations and policies	6.4	6.5	Low Priority
a8	Coworkers	6.3	6.4	Low Priority
a9	Job nature	7.0	6.8	Keep up the Good Work
a10	Communication	6.6	6.2	Low Priority
Mean		6.88	6.64	

The aforementioned IPA results revealed the importance and performance levels of the 10 criteria. The DEMATEL method was subsequently conducted to examine the causal relationships and degrees of influence between these criteria.

### DEMATEL results

(1) Expert opinions

Table 2 presents a summary of the expert opinions obtained through the DEMATEL questionnaire. The scores assigned by the 15 experts were averaged to one decimal place to yield a 10-criterion matrix comprising 100 grids. After 10 diagonal grids that represented zero influence were removed, the remaining 90 grids representing various degrees of interactive influence of the criteria were obtained.

**Table 2:** Initial direct-relation matrix *X*.

Criteria	a1	a2	a3	a4	a5	a6	a7	a8	a9	a10
a1	0	5.2	6.8	7.8	6.8	3.2	4.1	2.4	7.3	5.2
a2	7.4	0	6.2	6.1	8.2	2.8	5.8	4.2	7.3	3.7
a3	4.8	2.7	0	6.6	4.3	0.8	4.1	1.7	4.7	3.8
a4	4.4	1.9	4.9	0	0.7	0.8	0	0	1.4	1.0
a5	0	0	1.2	3.3	0	1.8	0.9	3.4	0	0
a6	1.8	1.9	2.3	1.9	0	0	0	0	2.7	2.2
a7	0	0	2.1	0	0.9	0	0	1.2	0	0
a8	0	0	2.4	0	2.3	0	0	0	1.2	1.7
a9	8.2	7.9	7.8	8.2	5.2	2.8	4.3	4.1	0	4.9
a10	0	0	4.7	3.3	4.1	2.1	2.4	1.7	2.8	0

(2) Estimation results

Next, the direct-relation matrix was normalized; column vectors with maximal values were used as normalization baselines. The reciprocal of the maximal value of the summed columns was the *λ* value. Eq 2 was used to multiply the direct-relation matrix *X* by *λ* to derive the normalized direct-relation matrix *N*. The influential coefficients were then rounded to two decimal places (Table 3).

**Table 3:** Normalized direct-relation matrix *T*.

Criteria	a1	a2	a3	a4	a5	a6	a7	a8	a9	a10
a1	0.00	0.10	0.13	0.15	0.13	0.06	0.08	0.04	0.14	0.10
a2	0.14	0.00	0.12	0.11	0.15	0.05	0.11	0.08	0.14	0.07
a3	0.09	0.05	0.00	0.12	0.08	0.01	0.08	0.03	0.09	0.07
a4	0.08	0.04	0.09	0.00	0.01	0.01	0.00	0.00	0.03	0.02
a5	0.00	0.00	0.02	0.06	0.00	0.03	0.02	0.06	0.00	0.00
a6	0.03	0.04	0.04	0.04	0.00	0.00	0.00	0.00	0.05	0.04
a7	0.00	0.00	0.04	0.00	0.02	0.00	0.00	0.02	0.00	0.00
a8	0.00	0.00	0.04	0.00	0.04	0.00	0.00	0.00	0.02	0.03
a9	0.15	0.15	0.15	0.15	0.10	0.05	0.08	0.08	0.00	0.09
a10	0.00	0.00	0.09	0.06	0.08	0.04	0.04	0.03	0.05	0.00

Eqs 3 and 4 were subsequently employed to obtain the total-relation criteria matrix T (Table 4).

**Table 4:** Total-relation criteria matrix *T*_*C*_.

Criteria	a1	a2	a3	a4	a5	a6	a7	a8	a9	a10	*Di*
a1	0.11	0.17	0.25	0.27	0.23	0.11	0.15	0.11	0.22	0.17	1.80
a2	0.23	0.08	0.25	0.25	0.26	0.10	0.18	0.15	0.22	0.15	1.88
a3	0.15	0.10	0.10	0.21	0.16	0.05	0.13	0.08	0.15	0.12	1.25
a4	0.12	0.07	0.14	0.06	0.06	0.04	0.04	0.03	0.07	0.06	0.69
a5	0.01	0.01	0.04	0.07	0.01	0.04	0.02	0.07	0.01	0.01	0.31
a6	0.07	0.06	0.09	0.08	0.04	0.02	0.03	0.02	0.08	0.07	0.58
a7	0.01	0.00	0.05	0.01	0.02	0.00	0.01	0.03	0.01	0.01	0.14
a8	0.01	0.01	0.06	0.02	0.06	0.01	0.01	0.01	0.03	0.04	0.28
a9	0.26	0.22	0.29	0.30	0.22	0.11	0.17	0.15	0.12	0.18	2.00
a10	0.04	0.03	0.13	0.11	0.11	0.06	0.07	0.06	0.08	0.03	0.71
*Rj*	1.02	0.75	1.40	1.40	1.19	0.54	0.81	0.70	1.00	0.83	

**Fig 2:**
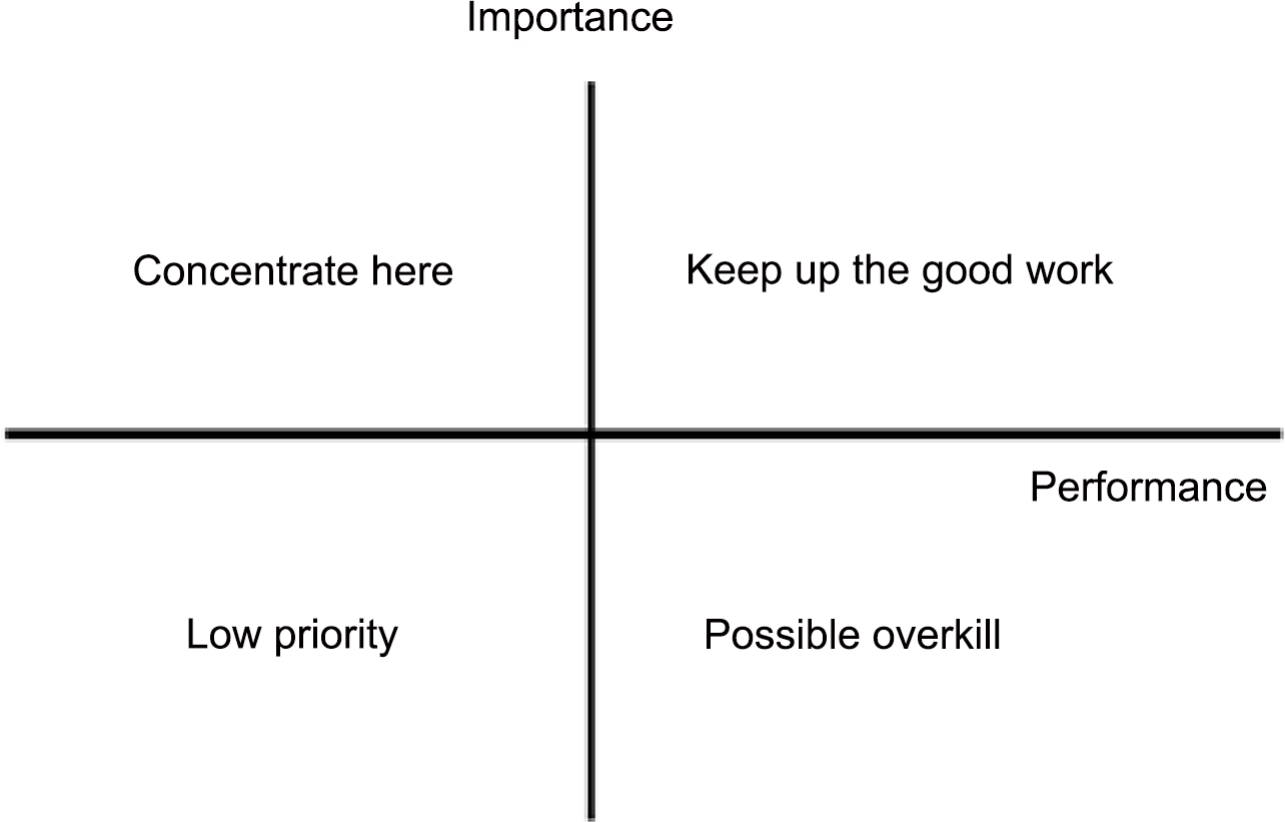
Interactive influence among the 10 criteria.

Finally, the value of each column (*D*_*i*_) and row (*R*_*j*_) was calculated using Eqs 5 and 6 to obtain the prominence (*D + R*) and relation (*D*–*R*), as shown in Table 5. In addition, the 10 criteria were plotted with prominence on the x-axis and relation on the y-axis (Fig 2).

**Table 5:** Prominence and relation results obtained through DEMATEL.

Criteria	*D*	*R*	*D + R*	*D—R*
a1	1.80	1.02	2.81	0.78
a2	1.88	0.75	2.63	1.13
a3	1.25	1.40	2.65	-0.15
a4	0.69	1.40	2.09	-0.71
a5	0.31	1.19	1.50	-0.88
a6	0.58	0.54	1.11	0.04
a7	0.14	0.81	0.95	-0.67
a8	0.28	0.70	0.98	-0.42
a9	2.00	1.00	3.00	1.00
a10	0.71	0.83	1.55	-0.12
Mean			1.93	0.00

Based on the analysis results presented in Table 5 and Fig 2, the degrees of influence and the causal relationships among the 10 R&D personnel satisfaction criteria are described as follows:

1. High relation, high prominence: compensation (a1), promotion (a2), and job nature (a9). These three criteria were “cause” criteria, which were the core items that influenced other criteria. Thus, they were the driving factors for problem solving.
2. High relation, low prominence: work environment (a6). This criterion minimally influenced a few other attributes, indicating that it was relatively independent.
3. Low relation, low prominence: recognition (a5), regulations and policies (a7), coworkers (a8), and communication (a10). These criteria were slightly influenced by the others, suggesting that they were relatively independent.
4. Low relation, high prominence: supervisors (a3) and fringe benefits (a4). These two criteria were “effect” criteria that were influenced by other criteria. Although they required improvement, a3 and a4 could not be directly improved because they were effect criteria.

In summary, among the 10 R&D personnel job satisfaction criteria, compensation (a1), promotion (a2), and job nature (a9) were determined to lie in the high-relation, high-prominence quadrant, indicating that they were the core items influencing the other criteria. Thus, improving performance pertaining to these three criteria may contribute toward solving core problems while enhancing performance associated with other criteria.

## Discussion

After an analysis of the IPA and DEMATEL results, the results of both models were integrated to derive managerial implications from the analysis of the 10 R&D personnel job satisfaction criteria.

Based on the IPA results, promotion (a2), work environment (a6), and job nature (a9) attained high satisfaction and belonged to the Keep up the Good Work quadrant. After the IPA and DEMATEL results were combined, promotion (a2) and job nature (a9) were located in the high-relation, high-prominence quadrant and categorized as cause criteria. Thus, the LCD industry should continue to improve both criteria and retain their lead in R&D personnel job satisfaction, thereby making professionals more satisfied at the workplace and enhancing the performance of other criteria. After the results were integrated, work environment (a6) lay in the high-relation, low-prominence quadrant, indicating that this criterion slightly influenced a few other criteria and exhibited relative independence.

The IPA results showed that compensation (a1) and fringe benefits (a4) fell in the Concentrate Here quadrant. After the IPA and DEMATEL results were combined, compensation (a1) was located in the high-relation, high-prominence quadrant and categorized as a cause criterion that was a core item affecting other criteria and a driving factor for problem solving. Based on these results, LCD companies should focus their resources on R&D personnel dissatisfaction by focusing on compensation problems. Otherwise, their low job satisfaction will not improve. Fringe benefits (a4) lay in the low-relation, high prominence quadrant. This criterion was affected by others, and although it required improvement, it was an effect criterion that could not be directly improved.

The IPA results placed regulations and policies (a7), coworkers (a8), and communication (a10) in the Low Priority quadrant. After the IPA and DEMATEL results were combined, these three criteria fell in the low-relation, low-prominence quadrant. They were slightly affected by other criteria, indicating their relative independence.

From the IPA results, supervisors (a3) and recognition (a5) were located in the Possible Overkill quadrant. After the IPA and DEMATEL results were combined, supervisors (a3) lay in the low-relation, high-prominence quadrant and recognition (a5) in the low-relation, low-prominence one. Both criteria had relative dependence, receiving slight influence from others and exerting slight influence.

High-tech industry competitiveness relies on R&D professionals. Accordingly, the widespread shortage of R&D employees hinders the development of high-tech industries. R&D personnel job satisfaction can be improved as a possible solution to this industrial challenge. Based on the findings regarding the criteria for evaluating the job satisfaction of R&D professionals in the LCD industry, three criteria were determined to be essential for improving job satisfaction. This study observed good performance of promotion (a2) and job nature (a9) in the industry and recommends that they be considered key criteria and consistently improved to maintain high job satisfaction among R&D personnel. However, compensation (a) attained poor performance and required improvement. The industry should address issues concerning R&D personnel’s compensation to improve their overall job satisfaction, because expending excessive resources on other criteria may yield only limited results.

## Conclusion

The growth of a company depends on the competitiveness and capability of its R&D personnel. The process of R&D involves considerable labor and resources. Moreover, the knowledge, skills, attitudes, and innovative capability of R&D professionals lay the foundation for industrial and social progress and determine the success of a business.

This study identified two limitations of the employee satisfaction criteria adopted by previous studies. First, the importance and performance of each criterion have not been estimated in some studies; thus, their relevance could not be measured. Second, most studies have assumed that these criteria are independent of each other and no interactive influence or causal relationship exists between them. This assumption may limit the improvement of employee satisfaction criteria. To address both limitations, this study integrated IPA and the DEMATEL method to develop an IPA–fuzzy DEMATEL model. IPA was conducted first to measure the importance of and satisfaction gained from all job satisfaction criteria. The analysis of the DEMATEL model was then used to determine the causal relationships and degrees of influence among the criteria. Finally, a criteria model was constructed to evaluate the job satisfaction of high-tech R&D personnel.

We determined that compensation (a1), promotion (a2), and job nature (a9) are the main factors for problem solving. Thus, improving satisfaction associated with these three criteria can enhance satisfaction associated with other criteria. In addition, promotion (a2) and job nature (a9) have good performance, which LCD companies should continue to maintain. However, compensation (a1) has poor performance and should be improved. Managers should place more emphasis on these three criteria to increase job satisfaction among R&D personnel, thereby retaining or attracting more of such professionals.

## Limitations and Suggestions

This study examined only one industry as a case to verify the self-designed criteria model for evaluating R&D personnel job satisfaction. Future research should investigate the job satisfaction of R&D professionals in other industries. Moreover, other criteria methods can be applied to explore R&D professionals’ job satisfaction and compare the results with those obtained with the criteria proposed in this study.
